# The Impact of Hydrogen on Mechanical Properties; A New In Situ Nanoindentation Testing Method

**DOI:** 10.3390/mi10020114

**Published:** 2019-02-11

**Authors:** Christian Müller, Mohammad Zamanzade, Christian Motz

**Affiliations:** 1 Department of Materials Science and Methods, Saarland University, Bldg. D2.2, 66123 Saarbrücken, Germany; c.mueller@matsci.uni-saarland.de (C.M.); motz@matsci.uni-sb.de (C.M.); 2 Mines Saint-Etienne, Univ Lyon, CNRS, UMR 5307LGF, Centre SMS, F-42023 Saint-Etienne, France

**Keywords:** nickel, nanoindentation, hardness, brittleness and ductility, hydrogen embrittlement

## Abstract

We have designed a new method for electrochemical hydrogen charging which allows us to charge very thin coarse-grained specimens from the bottom and perform nanomechanical testing on the top. As the average grain diameter is larger than the thickness of the sample, this setup allows us to efficiently evaluate the mechanical properties of multiple single crystals with similar electrochemical conditions. Another important advantage is that the top surface is not affected by corrosion by the electrolyte. The nanoindentation results show that hydrogen reduces the activation energy for homogenous dislocation nucleation by approximately 15–20% in a (001) grain. The elastic modulus also was observed to be reduced by the same amount. The hardness increased by approximately 4%, as determined by load-displacement curves and residual imprint analysis.

## 1. Introduction

Conventional mechanical testing methods have been used for quantitative studies of the influence of hydrogen on mechanical properties, e.g., yield stress, ultimate tensile stress and fracture strain [1]. However, these techniques are not very successful in obtaining mechanistic information because they simultaneously probe a large volume of the material and only provide an averaged result as if the volume were homogeneous. In fact, macroscopic samples contain inhomogeneities such as vacancies, dislocations and grain boundaries, which are known to play an important role in hydrogen embrittlement [2,3,4]. In contrast to macroscopic experiments, local testing methods enable us to decrease the probed volume of material, perform measurements for a quasi-homogeneous volume of material and hence decrease the possible sources of scattering in the results [5,6,7]. Additionally, as a result of the small probed volume, the hydrogen concentration can be assumed to be constant. In the past, we have used various local, in situ experimental techniques, such as electrochemical nanoindentation, in situ compression and bending of micro-pillars, to study the contribution of solute hydrogen on elastic properties, dislocation nucleation and hardness of alloys. These techniques enabled us to achieve an understanding of the mechanisms of hydrogen embrittlement (HE) for a material in a certain medium. Furthermore, we were able to rank the sensitivity of different alloys to hydrogen embrittlement in a specific medium irrespective of the impacts of grain boundaries, phase boundaries, pores, etc. [5,6,7].

However, there is still room for improvement to make electrochemical setups easier and experiments more reproducible. Our previous in situ nanoindentation approach comprised a layer of electrolyte on the specimen surface, which was penetrated by the tip during indentation. This had several disadvantages: (i) capillary forces acting on the tip; (ii) an inability to use the optical microscope of the machine for positioning; (iii) either an electrolyte flow causing vibrations or a static electrolyte film, in which no chemical reaction products are washed away from the surface; (iv) corrosion of the tested surface and, accordingly, (v) limited time for hydrogen charging as well as mechanical testing. In this paper, a new testing setup for studying the impact of hydrogen on mechanical properties is introduced. With this method, hydrogen is provided at the bottom surface of a thin sample while nanoindentation is performed on the top surface. This method avoids most of the problems named above and also allows the analysis of previously unobtainable properties with respect to diffusion. 

## 2. Experiments

**Figure 1:**
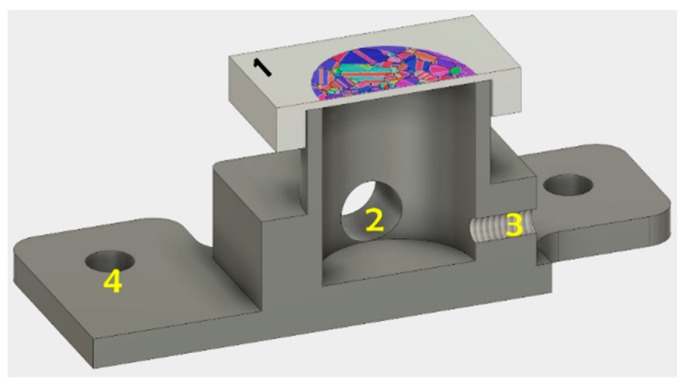
Cross section of specimen and electrochemical cell. (1) Nickel specimen, (2) electrolyte in-/output, (3) screw as counter electrode (4) holes for fixation in the nanoindenter.

Pure (99.9%) polycrystalline, square-shaped nickel specimens were milled from the back, heat-treated at 1240 °C for grain growth, ground in multiple steps, and finally electropolished with a solution of 1M sulfuric acid solved in methanol. These steps minimized the possible amount of residual stress and plastic deformation in the material, especially near the surface, where no sources for inhomogeneous dislocation nucleation were desired. The resulting specimen geometry had a thickness of approximately 200 μm in the region of interest (marked as the area with superimposed electron backscatter diffraction (EBSD) map in Figure 1), which was smaller than the average grain diameter (~500 μm). The sample was then glued to the dedicated cell made from polyvinyl chloride (PVC). The holes in the base plate of the cell allowed us to securely install it in the indenter, ensuring it was positioned identically before and after hydrogen permeation. The detailed specimen preparation and configuration of the electrochemical setup have been published elsewhere [8].

The first electrochemical hydrogen charging step was carried out ex situ for two days with a constant current density of 14.5 A/m², applied by an IVIUM CompactStat (Ivium Technologies B.V., Eindhoven, The Netherlands). A 0.25 molar H_2_SO_4_ solution was used as the electrolyte, containing 5 g/L potassium iodide (KI) as hydrogen recombination poison. To remove hydrogen bubbles at the charging surface, the sample was inverted and the solution was steadily pumped in and out. The risk of outgassing was accounted for by charging in situ during measurements. For this purpose, a lower current density of 0.25 A/m² was applied and the flow rate of the pumped solution was decreased to avoid vibrations. Furthermore, a borate buffer solution (mixed using 1.24 g/L H_3_BO_3_ and 1.91 g/L Na_2_B_4_O_7_·10H_2_O, also supplemented with 5 g/L KI) was used instead of sulfuric acid. However, the outgassing of hydrogen may also be considerably reduced by the existence of a homogeneous passive layer [8,9], which was measured to be approximately 10 nm, using a JEOL JEM-ARM200F transmission electron microscope (TEM). 

As a proof of concept, a (001)-oriented grain was tested before and after hydrogen charging. Nanoindentation was performed with a Hysitron Triboindenter (Bruker Corporation, Billerica, MA, USA), equipped with Performech controller and a diamond Berkovich tip with a tip radius of approximately 400 nm. The applied indentation parameters are summarized in Table 1, where “fast” and “slow” corresponds to the loading rate.

## 3. Results and Discussions

We observed an elastic deformation of the thin membrane during nanoindentation despite the low applied forces. Quantitative analysis of the results was performed after precise evaluation of total system compliance, which can be interpreted as a series connection of three springs: (i) compliance of the machine frame, (ii) PVC cell and the glues used to install the sample, and (iii) the deflection of the thin nickel membrane. The first calibration was a standard calibration involving the indentation of a fused quartz reference with high forces. The second spring constant was evaluated by testing a bulky nickel sample installed on the same cell and attached with the same glue. To evaluate the third spring constant or the effect of the bending of the whole membrane on the nanoindentation curves, the continuous stiffness method (Hysitron NanoDMA measurement) was used. This technique measures the elastic modulus at every indentation depth by continuously oscillating the tip. On a reference specimen, we verified that the modulus of the nickel bulk is independent of depth. Hence, the internal compliance value in all other data files were modified until their NanoDMA results also met this requirement.

**Figure 2:**
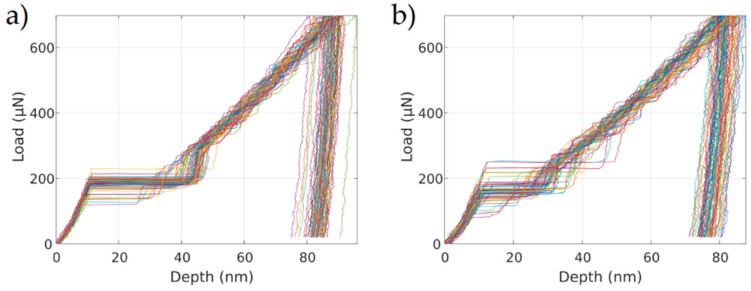
Load-displacement curves recorded with the fast loading rate (**a**) before charging and (**b**) after charging with hydrogen. Curves in which no pop-in could be detected were filtered.

Figure 2 shows load-displacement curves before and after charging. The pop-in or displacement burst phenomenon was related to the nucleation of dislocations around maximum shear stress under the tip [10]. The probability of the pop-in event did not change after hydrogen charging (in both cases more than 95%). The few curves in which no distinct pop-in could be detected are not displayed. A 15% reduction of average $P_{\mathrm{pop}-\mathrm{in}}$ values was determined to be attributable to hydrogen charging as well as a slight increase in scattering. The scattering in $P_{\mathrm{pop}-\mathrm{in}}$ is a common observation in nanoindentation experiments, originating from the thermal activation of homogenous dislocation nucleation [11,12].

Results indicate that the critical energy needed for dislocation nucleation was decreased, which agrees with previous studies [5,7,13]. Because of the slight reduction of the average values of pop-in load and because the population of the pop-in event did not change after charging, we can assume that the dislocation nucleation was homogeneous, and the observed behavior could be related to the debonding effect of hydrogen, known as hydrogen-enhanced decohesion (HEDE). 

**Figure 3:**
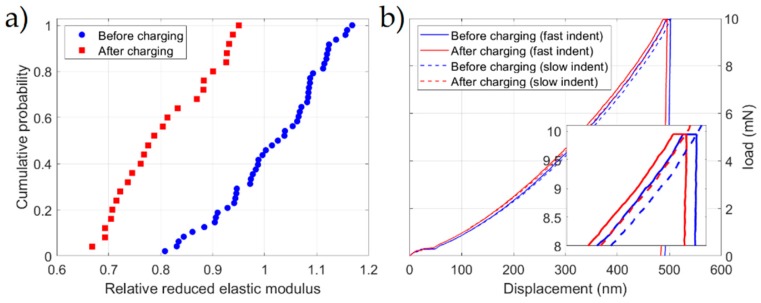
(**a**) Plot of reduced elastic modulus determined from Hertzian loading. (**b**) Load-displacement data of hardness measurements (averaged across 10 indents).

Another consequence of decohesion is the reduction of the elastic modulus, which was noticeable as a reduction of the slope in the Hertzian regimes, where the curves follow the equation [14]:

(1) $$P=\frac{4}{3}E_{r}\sqrt{R}\times h^{3/2}$$

in which $E_{r}$ is the reduced elastic modulus, R is the tip radius, P. is the applied load, and h the indentation depth. The load-displacement (P−h) data before the pop-in can be fitted to this equation with a fit parameter proportional to $E_{r}$. To visualize the differences and make them independent of R, a cumulative distribution of relative elastic moduli $E_{r}/E_{0}$ is plotted in Figure 3a, where $E_{0}$ is the average value before hydrogen charging. The reduction of reduced elastic modulus according to this analysis is approximately 17±10%, from 206±20 GPa to 171±17 GPa. Both individual values are in a realistic range for nickel. The reliability of each data point obtained by curve fitting and the deviation between individual points account for the uncertainty of this result. A more conventional method to determine Young’s modulus in nanoindentation experiments is to fit the unloading segment with the model introduced by Oliver and Pharr [15]. Using this approach for our measurements, the moduli before and after charging were both approximately 200 GPa and were well inside the statistical error interval of each other. 

The occurrence of a measurable difference in Young’s moduli due to hydrogen is controversial. For example, Lawrence et al. determined a comparable reduction by about 22% in nanoindentation experiments on nickel with the conventional Oliver-Pharr method [16]. In other studies, the difference was much smaller or even negligible, e.g., in molecular dynamics simulations of hydrogen in nickel [17]. Nevertheless, the analysis of elastic moduli is very much possible in our setup after the previously mentioned compliance correction.

Using the reduction of both $P_{\mathrm{pop}-\mathrm{in}}$ and $E_{r}$, we can also calculate the reduction of the shear stress necessary for dislocation nucleation. According to the Hertzian model, the maximum shear stress under the tip is given by:

(2) $$\tau_{\max}=0.31\times {\left(\frac{6PE_{r}{}^{2}}{\pi^{3}R^{2}}\right)}^{1/3}$$

in which both P and $E_{r}$ are reduced by 15–17%, so the stress decreases by the same amount. The calculated values of approximately 5–7 GPa are close to the expected theoretical shear strength of nickel [18], which confirms that the cause of the pop-in was indeed homogenous dislocation nucleation. 

Figure 3b shows the P-h curves performed to study the impacts of strain rates and dissolved hydrogen on the plastic behavior of Ni. Each curve is an average of ten indents, which was calculated and plotted. Similar to other solute atoms, hydrogen can contribute to the pinning of dislocations by forming Cottrell atmospheres around dislocation cores [19,20]. The resulting reduction of dislocation mobility becomes clear if we compare the hardness before and after hydrogen charging. Measurements after hydrogen charging systematically show a decreased indentation depth at the maximum load. Increasing the loading rate also results in a smaller depth and therefore higher hardness, but this effect appears to be independent of hydrogen concentration. After measuring the projected area of the residual imprint of every indent, we determined a 4.3% increase in hardness due to hydrogen charging. Our results indicate that the application of various strain rates does not change this pining effect. Accordingly, the dislocations are constantly aged to saturation at room temperature at both tested strain rates. This means that when a dislocation escapes from a pinning point, the hydrogen diffusion is fast enough (relative to the dislocation velocity) to immediately follow and pin it again.

**Figure 4:**
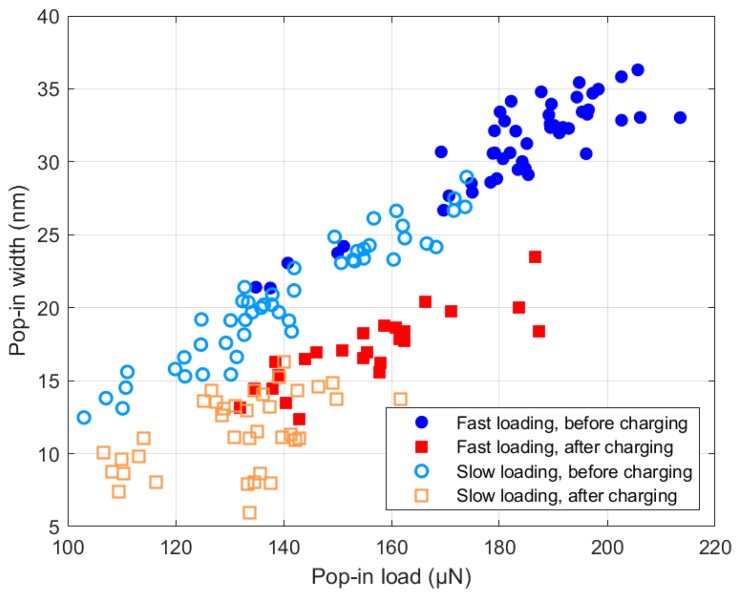
The impact of loading rate and hydrogen on the pop-in width and pop-in load.

Another observation that reinforces the proposition of reduced dislocation mobility is a substantial reduction of pop-in width (also called excursion length) after hydrogen charging. As Figure 4 shows, a pop-in that happened at the same load P would, on average, cause a much smaller excursion length. This is attributable to the dislocations which show a drag force caused by hydrogen, decelerating the tip so that it stops earlier. Although excursion length is often assumed to solely represent the number of nucleated dislocations [21], we believe that the gliding of dislocations plays an important role in the measured pop-in width. Therefore, although hydrogen can ease the nucleation of dislocations, the reduction of the slope of the curves in Figure 4 after hydrogen charging could be related to the sessile behavior of dislocations. 

## 4. Conclusions

The increase in hardness indicates a decreased mobility of dislocations due to the solute drag effect of hydrogen. However, hydrogen charging reduces the elastic modulus and the pop-in load and, accordingly, facilitates the formation of dislocations. The tested specimen geometry and charging conditions have proven to be successful and may be able to further analyze the influences of grain orientations and grain boundaries resulting from the testing of a thin layer charged from the back. The concept is promising for future research on diffusion coupled with changes in the mechanical properties of a variety of conductive materials.

One disadvantage introduced by the proposed setup is the consequence of the thin specimen geometry. The coarse-grained nickel layer is prone to deformation. Although its influence could be easily eliminated from nanoindentation curves, it leads to a long preparation procedure in which care must be taken in every step. Some of the electrochemical uncertainties also remain: The bottom of the sample may still be exposed to corrosion and the local hydrogen concentration at a certain point on the top can depend on inhomogeneities through which the hydrogen has to diffuse. In combination with potential discrepancies in delays between ex situ and in situ charging (and therefore outgassing), this remains a limitation on the quantitative reproducibility of specific results.

## Figures and Tables

**Table 1:** Nanoindentation parameters.

Measured Parameter	Max. Load	Load or Strain Rate	Type of Test	# of Indents
Pop-in slow	700 µN	50 µN·s^−1^	Quasi static	60
Pop-in fast	700 µN	5000 µN·s^−1^	Quasi static	60
Hardness fast	10 mN	0.5 s^−1^	Quasi static	10
Hardness slow	10 mN	0.05 s^−1^	Dynamic NanoDMAIII	10
